# What will mobile and virtual work look like in the future?—Results of a Delphi-based study

**DOI:** 10.1007/s11612-022-00627-8

**Published:** 2022-04-29

**Authors:** Simone Kauffeld, Darien Tartler, Hendrik Gräfe, Ann-Kathrin Windmann, Nils Christian Sauer

**Affiliations:** 1 Department for Work, Organizational and Social Psychology, Technische Universität of Braunschweig, Braunschweig, Germany; 2 University in the Academy of the Police Hamburg, Hamburg, Germany

**Keywords:** Mobile work, Virtual collaboration, Work design, Future of work, Mobile Arbeit, Virtuelle Zusammenarbeit, Arbeitsgestaltung, Zukunft der Arbeit

## Abstract

This contribution to the journal “Group. Interaction. Organization.” (GIO) takes a closer look at the mobile work of the future. Mobile work as well as virtual collaboration is becoming more and more integrated into our everyday working lives—not least reinforced by the COVID-19 Pandemic. In a Delphi-based study, we investigated the question of what mobile and virtual work will look like in the future. For this purpose, 35 future scenarios were described or processed in four rounds with a total of 460 experts and their desirability and probability of occurrence were evaluated. Positive developments are expected for the organization in terms of technology (e.g., advances in virtuality and artificial intelligence), leadership (e.g., increase in shared leadership and participation) and the work-life integration of employees (e.g., more flexibility and self-management). Negative effects are seen for teamwork (e.g., it becomes more difficult to build and maintain team cohesion and social exchange). How the challenges can be dealt with in terms of work design is shown and discussed.

## Abstract (de)

Dieser Beitrag der Zeitschrift „Gruppe. Interaktion. Organisation.“ (GIO) setzt sich genauer mit der mobilen Arbeit der Zukunft auseinander. Mobile Arbeit sowie virtuelle Zusammenarbeit wird verstärkt durch die Corona Pandemie immer mehr in unseren Arbeitsalltag integriert. In einer Delphi-basierten Studie haben wir uns mit der Frage auseinandergesetzt, wie die mobile und virtuelle Arbeit der Zukunft aussehen wird. Dazu wurden in vier Runden mit insgesamt 460 Expert*innen 35 Zukunftsszenarien beschrieben bzw. bearbeitet und deren Erwünschtheit und Eintretenswahrscheinlichkeit bewertet. Positive Entwicklungen werden für die Organisation hinsichtlich Technologie (z. B. Fortschritte in der Virtualität und der künstlichen Intelligenz), Führung (z. B. Zunahme der geteilten Führung und Beteiligung) und die Work-Life-Integration der Mitarbeitenden (z. B. mehr Flexibilität und Selbstmanagement) erwartet. Negative Auswirkungen werden dagegen für die Teamarbeit gesehen (z. B. wird es schwieriger, Teamzusammenhalt und sozialen Austausch aufzubauen und aufrechtzuerhalten). Es wird aufgezeigt und diskutiert, wie mit den Herausforderungen arbeitsgestalterisch umgegangen werden kann.

## Introduction

The onset and subsequent spread of the COVID-19 pandemic suddenly and drastically changed the working circumstances of millions of employees, as mandated social distancing, imposed as part of the effort to control the rising rate of infection, caused a record number of employees to work from home (Wandt 2020). Employment had already been undergoing fundamental changes prior to this disruptive event, fueled by the accelerated development of technology and the associated digital transformation (Kunze et al. 2020). The ongoing transformation has caused work to become increasingly agile while focusing on self-organization and customer-orientation (Neumer and Nicklich 2021). Along with the evolving work environment, employees’ expectations regarding flexible working conditions, participation, and autonomy have changed as well (Kauffeld and Sauer 2019).

Even before the COVID-19 pandemic, many employees supported the idea of remote work (Mergener 2020). However, employers’ concerns served as a roadblock to the execution of this concept. Managers primarily associated mobile work with a loss of control, including delayed feedback and technical difficulties. Among further managerial concerns were a higher potential for conflicts and unfair treatment among co-workers (Boos et al. 2017). On the organizational level, the culture of presence and emphasis on results added to the negative perception (Shockley and Allen 2010). Consequently, only about 12–25% of employees occasionally worked from home before the pandemic (Grunau et al. 2019). Along similar lines, the Braunschweig study on mobile work showed that less than 20% of time on the job was spent working remotely (Kauffeld 2020).

The COVID-19 pandemic can be seen as a catalyst for the consideration and implementation of mobile work in organizations, as it has drastically accelerated the shift from face-to-face to virtual teamwork (Kniffin et al. 2021). Again, drawing upon the Braunschweig study, in April 2020, about 85% of local employees worked remotely, with 60% working exclusively from home (Kauffeld 2020). The latter figure stabilized at 40% over the subsequent survey periods. During the same time frame in Germany, 26% of German workers were entirely working from home, while 35% combined work from home (WFH) and on-site work (Grabka et al. 2020; Möhring et al. 2020). Most strikingly, the results encompass fields of work (e.g., public administration, health insurance, education) in which presence was considered indispensable prior to the pandemic as well as industries with a focus on virtual collaboration (e.g., IT, research).

The radical changes at work have also led to a significant change in attitude. As a result, employees’ calls for increased opportunities for mobile work have grown louder (Kunze et al. 2020). Since the general return to full-time face-to-face work in the fall of 2020, a significant majority of workers have advocated for the possibility to work from home for 2 or 3 days per week (Wandt 2020). In support of this development, in a recent survey, 56% of employees expressed a preference to work at least partially from home in the future (Kunze et al. 2020). This desire is particularly pronounced among women and parents (Arntz et al. 2020). In summary, a balanced mix of mobile and face-to-face work might represent an ideal prospective work model for most workers at the present time.

## Definition of mobile work

Mobile work is defined as employees performing their work outside the corporate office. It is characterized by the collaborative use of new information technologies as well as high flexibility and autonomy (i.e., Benz 2010; Brandt 2010). In contrast to location-based telework, mobile work can take place at any time and place, including on a train or in a café (Chudoba et al. 2005). The term “smart work” has been used for work that focuses on the use of smart devices (Lee and Lee 2012), allowing teams to communicate and coordinate with each other regardless of location. For example, a team member may conduct a virtual meeting from home, joined by another participant working from the office and a third from a train (Kauffeld 2020). Mobile work is enabled by modern information and communication technologies (ICT). These virtual tools go beyond e‑mail, chat, and video functions to include various management systems (e.g., knowledge and customer relationship management) and collaboration software that facilitate real-time access to shared information, synchronous communication, and virtual teamwork (Antoni and Syrek 2017; Kauffeld et al. 2016).

Prior to the COVID-19 pandemic, this type of virtual teamwork was primarily a topic for global corporations with teams and locations spread across several continents. However, the pandemic’s impact has shifted the focus to providing opportunities to work from home (Arntz et al. 2020; Handke and Kauffeld 2019). Some researchers have estimated that more than 30% of jobs in the United States could be performed remotely (Dingel and Neiman 2020). Similarly, Arntz et al. (2020) computed a teleworkability index for various occupations in Germany and found that about 31% of jobs could be carried out from home. In addition, the authors identified a further 12% of jobs containing a substantial proportion of tasks that could easily be performed remotely. The different tasks could thus be split up between days of on-site work and mobile workdays or divided over a single working day.

### Opportunities for mobile work

Opportunities for mobile work differ among organizations, teams, and employees (Akin and Rumpf 2014). Advantages vary among these distinct perspectives as well. Organizations can save wage costs, expenditures on business trips, and office space, to the point that regions with low infrastructure and lower office rents become attractive (Handke and Kauffeld 2019). Teams can be built solely based on members’ qualifications without the need to consider spatial distribution (Konradt and Hertel 2002). For employees, mobile work provides opportunities to carry out tasks independent of time and location. The increased flexibility offers the chance to better reconcile private and professional life and, thus, improve work–life balance. Tasks can be performed undisturbed and uninterrupted, facilitating concentrated work and the experience of flow. Less commuting reduces travel time and costs (e.g., Gilson et al. 2015). As a result, WFH has been linked with better time management and a more pleasant work atmosphere, which has a positive impact on absenteeism and work motivation (Kauffeld et al. 2016; Knieps and Pfaff 2020).

In 2015, Bloom and colleagues randomly allocated call center employees to work either from home or the office (Bloom 2015). According to the study’s findings, the workers who were more productive at home opted to keep working remotely on a permanent basis. Other recent experiments have confirmed that mobile work can increase workers’ productivity (Angelici and Profeta 2020). In a similar vein, employees who returned to full face-to-face work in the fall of 2020 reported higher levels of emotional exhaustion and reduced productivity (Wandt 2020). Overall, an increase in flexibility arguably facilitates employees’ self-determination. Research has also shown that greater flexibility increases autonomy and consideration of individual needs, which positively affects job satisfaction and job performance (Arntz et al. 2020). In this respect, the availability of a home office is seen as particularly beneficial for parents and mothers (Alon et al. 2020). As a consequence, researchers have postulated that the number of employees working from home will remain at a high level even after social distancing rules are lifted (Arntz et al. 2020; Boeri et al. 2020).

### Challenges of mobile work

Despite the vast number of advantages it offers, mobile work poses numerous challenges for organizations, teams, and employees (e.g., Mak and Kozlowski 2019). An increasing proportion of mobile work can negatively affect organizational identification as contacts within the company decline. Working from home frequently leads to the fear of career setbacks due to a lack of presence at the workplace and reduced involvement in decision-making processes (e.g., Ashforth 2020; Sohrabi et al. 2011). In most organizations, scholars have identified a lack of technical equipment and the prevalence of inadequate structures as the most problematic factors in dealing with the sudden change to remote work, which required transforming companies’ work procedures to virtual work environments on the fly (Carnevale and Hatak 2020; Kauffeld et al. 2016).

For teams, the abrupt transformation to mobile work and virtual collaboration has posed a challenge to team functioning due to necessary changes in crucial processes, such as coordination and communication (e.g., Latniak and Schäfer 2021; Powell et al. 2004). In a recent review, Handke et al. (2020) examined how work design shaped the impact of virtuality on teams and identified relevant work design characteristics for teams (i.e., interdependence, knowledge characteristics, job demands, and job resources). On the one hand, high levels of knowledge characteristics (e.g., task complexity or non-routineness) and job demands (e.g., role ambiguity or time pressure) were challenging for team functioning. On the other hand, job resources (e.g., feedback or social support) were positively related to team functioning, potentially by buffering the demands of virtual work. That said, the results of interdependency, and especially task interdependency, were mixed.

In addition, the sudden shift to mobile work presents a challenge for team leadership as well as team functioning. In virtual environments, managers must focus on a completely new set of demands since they are tasked with leading and motivating geographically dispersed team members (e.g., Bernardy et al. 2021; Hertel et al. 2005). Accordingly, task- and relation-oriented leadership behaviors have been identified as key factors for effective virtual leadership—especially in sudden crisis situations (Bartsch et al. 2021). In this light, managers must initiate virtual team structures, clarify expectations, coordinate task goals, and monitor work processes. Guidance on the manager’s part involves setting clearly defined goals and allocating roles for all team members (Judge et al. 2004; Hertel et al. 2005). That said, managers also need to facilitate collaborative interactions and foster a climate of social support (Mander et al. 2021). In this regard, creating the feeling of a collaborative and connected team while granting all team members autonomy in how they perform their tasks is essential (Liao 2017).

For employees, working from home results in the loss of daily face-to-face interactions among colleagues. This lack of social integration reduces feelings of social support, which scholars have considered a key factor to prevent procrastination, loneliness, and work–family conflict (Kniffin et al. 2021; Wang et al. 2021). Moreover, higher flexibility also harbors risks. For example, the workload is significantly increased due to feelings of constant availability and self-exploitation (Koroma et al. 2014). Further pressure is created by higher demands for self-organization and decision-making. These factors result in a lack of separation between work and private life. As a consequence, psychological diagnoses, such as burnout, have significantly increased in recent years (Kunze et al. 2020).

### Work design characteristics for mobile work

In the wake of the COVID-19 pandemic, mobile work is here to stay. Therefore, organizations need to find ways to effectively deal with the sudden changes and adapt their work procedures. In this regard, work design theory presumes that different work characteristics affect organizational results and workers’ attitudes (Parker and Wall 2001). Along these lines, Morgeson and Humphrey (2006) identified four key work characteristics. *Task characteristics* refer to factors that influence the execution and accomplishment of the task (e.g., autonomy, task variety, feedback from job). *Knowledge characteristics* describe specific features of the skills required of a person (e.g., range of expertise or job specialization). *Social characteristics* comprise interpersonal aspects, such as interactions and cooperation at work (e.g., interdependence, feedback from others). Lastly, *contextual characteristics* refer to employees’ working conditions, such as physical demands and ergonomics (Stegmann et al. 2010). Arguing the necessity to consider work design characteristics in order to shape the use of digital technologies to support workers’ job motivation, satisfaction, and performance, Parker and Grote (2020) recently defined five further sub-categories.

Effectively implementing mobile work requires the consideration of specific *task characteristics*. In this regard, Parker and Grote (2020) saw *job autonomy and control* as fundamental aspects that influence motivation, performance, and stress. The increasing use of mobile technology has led to an interesting duality between autonomy and control, which researchers have termed the “autonomy paradox” (e.g., Jarvenpaa and Lang 2005; Mazmanian et al. 2013). For example, ICT can be used to remotely monitor employees’ activities—to the point that “digital footprints” can reveal work patterns—and thus, it has become a powerful leadership tool (Leonardi 2021). However, strict monitoring has been found to cause anxiety at work and create stress due to the pressure of constant availability (Parker et al. 2020). Contrariwise, smart devices can empower employees to carry out their tasks with more freedom and fewer boundaries than has ever been the case. In this context, autonomy has been found as a key to job satisfaction in virtual teams (e.g., Golden 2007; Robert and You 2018). We argue accordingly that the category of *job autonomy and control* is too broad, as it combines aspects of flexible working and influences that affect the structuring of tasks and choice of method. Instead, we propose two sub-categories: *job autonomy*, focusing on decision-making as part of the work process, and *job control*, which is concerned with choices of where and when to work. As a second critical factor, Parker and Grote (2020) defined *job feedback. *In virtual teams, sharing feedback has a positive effect on productivity while additionally fostering a team’s well-being by enabling relationship-building communication (Geister et al. 2006). This practice also increases the visibility and transparency of team members’ activities and provides role clarity (Peñarroja et al. 2017).

In the context of* knowledge characteristics, *new qualifications and specialized knowledge are required for the effective use of ICT (Fréour et al. 2021; Pfeiffer 2018). Otherwise, remote work could result in a simplification of work activities, which might lead to less task variety, with a subsequent reduction in intrinsic motivation (Kunst 2019; Szalavetz 2021). Thus, Parker and Grote (2020) defined the category *skill variety and use*, which showcases the importance of organizations providing employees with the qualifications they require to enable them to perform highly complex tasks from home using ICT. Moreover, beyond the new demands placed on employees, mobile work presents new challenges for managers. As previously argued, virtual collaboration and dispersed teams create new demands for leaders. We therefore propose the extension of the work design categories to include another sub-category specifically focused on *leadership*.

#### Social characteristics

are another critical aspect of mobile work since the importance of interpersonal relationships in the workplace is undisputed (Grant and Parker 2009). Even though ICT enables new ways of interaction, studies have shown that virtual communication is mainly focused on task-related aspects. In contrast, the neglect of social or relational aspects can result in a reduced perception of social support. As stated earlier, social support is a key factor in preventing feelings of loneliness, isolation, and low job satisfaction (Barnes 2012; Monzani et al. 2014). Accordingly, Parker and Grote (2020) defined *social and relational aspects* as crucial for organizations to foster social exchange and social contact among co-workers who work remotely.

#### Contextual characteristics

include aspects of the work environment, which change with the shift to mobile work. A literature review on health and remote work revealed that the increased use of technologies was connected with higher screen time and more sedentary activities (Buomprisco et al. 2021). These results show the importance of functionally adequate furniture at home, such as a suitable desk and chair, to prevent the deterioration of ergonomics (e.g., Hardwig and Weißmann 2021; Johnson et al. 2020). Beyond these ergonomic aspects, Parker and Grote (2020) argued that the use of digital technologies creates additional physical efforts associated with high costs, which they combined into a fifth category, named *job demands*.

In the context of digital transformation, new technologies are seen as the main factor influencing job resources and job demands. This view supports the idea that it is crucial for organizations to provide adequate ICT to enable effective mobile work. We thus argue that considering the expected advancements in *technology* as a separate category in addition to the key work characteristics is essential.

### Research questions

Even though the digital transformation of the workplace was already a relevant topic in the world of work before the pandemic, COVID-19 has now accelerated its development. This sudden change, however, has also brought a great deal of uncertainty to organizations in light of the advantages (e.g., increased flexibility, change in working hours, introduction of new technologies) and disadvantages (e.g., unclear availability, difficult accessibility, loss of control by managers) that have accompanied the increased implementation of digital technologies in equal measures. The prevailing uncertainty makes it unclear how the working landscape will look in the future. For this reason, going beyond the theoretical literature and experimental research by assessing employees’ opinions and attitudes toward these ongoing changes is highly relevant. On that account, the scenarios developed and evaluated for this study provide a deeper insight into possible future work situations, forming the basis for an initial strategic orientation for employers and employees alike.

Hence, we used a three-step method based on the Delphi technique with an additional post-workshop to identify and evaluate work scenarios relevant to the future of work. We then classified these scenarios in relation to work design theory categories of work characteristics. Accordingly, we aim to answer the following research questions:

#### RQ1

What scenarios can be derived for the future of work after COVID-19?

#### RQ2

How desirable are the scenarios?

#### RQ3

What is the estimated probability for the scenarios in the year 2030, compared to the estimated probability in 2021?

#### RQ4

How do desirability and probability go along in the year 2030 compared to 2021?

#### RQ5

What relevant work characteristics arise when describing scenarios for the future of work?

#### RQ6

What is the desirability and probability for work characteristics in 2021 compared to 2030?

## Method

### The Delphi method

The Delphi method is a research technique with an extensive history in the humanities and social sciences (Häder and Häder 2000). Since then, it has increasingly been used in business, health, and environmental fields (e.g., Fletcher and Marchildon 2014; Gnatzy et al. 2011; Taylor 2020). Häder and Häder (2014) described the broad goal of the Delphi method as twofold: first, as focused on collecting group opinions and combining it with targeted feedback, and second, as a tool to investigate predictions about the future by enabling exchange among experts to create and evaluate future scenarios. Similarly, Taylor (2020) defined the Delphi method as “a process for gaining consensus through controlled feedback from a panel—a group made up of experts or individuals knowledgeable on the subject” (p. 12). The use of multiple standardized questionnaires creates a highly structured and controlled group communication process, aiming to obtain a more accurate assessment of future developments through the inclusion of diverse experts in comparison to merely assessing individual or group opinions (Rowe and Wright 1999). Notably, however, a variety of further developments to the Delphi method have emerged in addition to the classic technique, leading to difficulties in achieving a uniform objective or definition (Häder and Häder 2014).

Rowe et al. (1991) emphasized four characterizing aspects in the development of Delphi studies. *Anonymity of the process* describes the requirement not to allow social or peer pressure from other participants to influence the interviewed experts. Therefore, at no point during the study may anonymity be dissolved. *Controlled feedback* is another highly relevant aspect of the Delphi method. In order to enable participants to broaden their own perspective, other people’s opinions are fed back and reflected to them. Effective feedback can thus increase the accuracy of the assessment*. Statistical aggregation of group responses* provides a suitable means for such feedback. The expert assessments allow a quantitative view of scenarios, facilitating the evaluation and interpretation of the data. As a fourth aspect, Rowe et al. (1991) addressed *iteration*. In the Delphi method, assessment and feedback are regularly fed back to the experts in several successive sequences. This process allows the experts to have a dynamic opinion, whereby scenarios can be constantly re-evaluated through regular feedback.

### Procedure

**Fig. 1:**
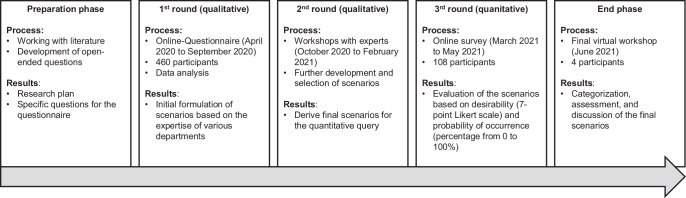
The Research Process. (Process is adapted from Häder and Häder 2014)

For our study, we adapted the classic Delphi process (e.g., Häder and Häder 2014) into a virtual context to make it more accessible during the COVID-19 pandemic. The process is divided into five work phases: the preparation phase, a first and second qualitative Delphi round, a quantitative third Delphi round, and a final expert workshop. Fig. 1 displays an overview of our adapted process.

The first step, the preparation phase, was specifically designed to allow us to become familiar with the Delphi method. In this phase, we outlined a detailed research plan, which included the necessary steps for the effective implementation of the survey. Following the classical Delphi process, open-ended questions were developed to be presented in a questionnaire to a diverse, comprehensive sample (Round 2).

The primary goal of the first and second Delphi rounds was to generate a variety of new ideas for the development of the scenarios. In the Delphi method, scenarios are defined for certain topics and phrased as easily understandable descriptions of a hypothetical but realistic future situation of the respective topic area (Hirsch-Kreinsen 2017). Scenarios often include several thematic aspects, making them more complex than conventional questionnaire items. According to Taylor (2020), open-ended questions offer a classic initial approach to developing scenarios in Delphi studies. In this context, open-ended questions enable a comprehensive inquiry into ideas, opinions, and information on a specific topic area in order to derive scenarios. In our study, we focused on the following main topic areas with open-ended questions:

- Professional challenges and risks (What are the professional challenges and risks you see in the COVID-19 pandemic?)
- Professional opportunities and possibilities (What are the professional opportunities and possibilities you see in the COVID-19 pandemic?)
- Private challenges and risks (What are the private challenges and risks you see in the COVID-19 pandemic?)
- Private opportunities and possibilities (What are the private opportunities and possibilities you see in the COVID-19 pandemic?)

Because the use of open-ended questions provides a large amount of content for deriving possible scenarios, this approach involves much work and evaluation for researchers. Thus, Taylor (2020) cited the possibility of literature review as a suitable alternative to idea generation with open-ended questions. To enable a comprehensive insight into possible future scenarios, we have combined a literature review with open-ended questions to derive and formulate initial scenarios in this study.

The aim of the second qualitative Delphi round was to confirm and finalize the previously derived future scenarios. To this end, six Delphi workshops were held, in which the participants were first presented with current research findings on remote work. Next, the previously collected scenarios were discussed. Since this round represented the second iteration, the developed scenarios were again revised and reselected following the discussions. For the final quantitative survey of scenarios in the third Delphi round, a total of 35 scenarios were selected and finalized. These scenarios were assigned to the work design categories adapted and extended from Parker and Grote (2020) by independent assessors. After a comprehensive discussion, two additional categories (leadership and technology) had to be added to optimally represent the scenarios, as leaving the relevant aspects in these two categories unaddressed would have made it difficult to unambiguously assign all scenarios a category. The agreement of the assessors was evaluated using the two-way random consistency intraclass correlation coefficient (ICC). The obtained ICC score of 0.89 indicated an excellent fit between the two assessors (Cicchetti 1994). The assignment of the scenarios to the categories was finalized in a collaborative workshop.

The aim of the quantitative third Delphi round was to evaluate the scenarios quantitatively according to desirability and probability of occurrence for 2021 and 2030. An online questionnaire used for this purpose included sociodemographic data (e.g., gender, age, occupation, leadership position) in addition to the 35 scenarios. For each scenario, participants were asked how desirable they considered the scenario to be (*D*), to what extent the scenario is already prevalent at this point in time (*EP2021*), and how prevalent they estimated it would be in 2030 (*EP2030*). Desirability was assessed on a 7-point response scale (−3 = not desirable, 0 = neutral, 3 = very desirable). Probabilities of occurrence were assessed in percentages ranging from 0% (not at all likely) to 100% (very likely). For each scenario, participants were also given an opportunity to describe their reasoning process.

The last round aimed at categorizing and evaluating the participants’ assessments. Following recommendations in the literature for a joint workshop to provide a better understanding of the assessment of the scenarios, the post-workshop was held with a select group of experts. In this workshop, the results of the quantitative online survey, including the participants’ reasoning behind their assessments, and the pre-selected scenarios were reported. In addition to the scenarios with the highest and lowest desirability and probability values, the scenarios with the highest disagreement in the evaluations were chosen. The workshop was held virtually due to COVID-19 constraints. The chosen scenarios were individually discussed and processed in depth. For each of the selected scenarios, the results of the discussion were recorded.

### Participants

The sample for the first and second Delphi rounds will be addressed only briefly, as it is primarily the sample for the third round that is relevant to this study. For the first Delphi round, a total of 460 people from different organizations were asked open-ended questions about the risks and opportunities they saw in the future of mobile work. The workshops from the second Delphi round took place in several organization-internal plenary sessions, which is why no participant information could be collected. The final scenarios for the third Delphi round were derived from the open-ended questionnaire responses and the information from the plenary rounds.

The sample for the third round was collected as a part of a longitudinal research project on how working from home due to the COVID-19 pandemic affects employees’ working life. The research project consists of an online questionnaire that was sent out virtually in March 2021. The following conditions had to be met for participation in the study: Prospective participants had to work at least partially from home and for a minimum of 20 h a week. Participation in the study was completely anonymous and voluntary. In total, we included 108 participants in our analyses. Slightly more participants identified themselves as female (55%) than male (45% male). On average, participants were 46 years of age, *SD* = 10.57, *R* = 25–63 years, with most of the participants having a university degree (66%). The three largest fields of work consisted of education (20%), the automotive industry (15%), and insurance (12%). In our sample, 33% of the participants held a leadership position. More than 58% of our sample worked in organizations with more than 1000 employees. Using the snowball sampling method, we disseminated the questionnaire in an untargeted manner via online and print media. This procedure was chosen to achieve a highly diverse sample since diversity is crucial in Delphi studies to obtain a multi-perspective view on the various scenarios (Förster and von der Gracht 2014).

The workshop for the final Delphi round included four participants who were familiar with the scenarios. Achieving a highly diverse sample was once again a focus in the selection of participants. As a result, the workshop featured an equal number of women and men. Moreover, people from the management and employee levels were brought together. The diversity aspect was additionally strengthened by the fact that the experts were people from research and practice, consequently making them able to bring different perspectives into the workshop. Lastly, the participants themselves had experience working from home, meaning that both an application-oriented perspective based on experience and a research-oriented view could be integrated into the workshop results.

## Results

### Scenarios for the future of work

The aim of this Delphi-based study was to describe and evaluate developments in mobile work and virtual collaboration. RQ1 focused on work scenarios that could be derived for the future of work. Qualitative data were provided by interviews and feedback from longitudinal online surveys. The data were revised and discussed with various experts as part of an iterative procedure. In the end, 35 scenarios were found, as shown in Table 1.

**Table 1:** Descriptive Statistics for the Derived Scenarios

Scenario	Dimensions		*n*	*M*	*SD*	Median	IQR
26 Meetings rarely take place exclusively on site anymore. It must always be possible to add people to a meeting virtually so that all the necessary people can take part in a meeting	JAC(2); Tech	D	93	2.04	1.4	2	–
		EP 2030	93	**85.23**	15.67	**90**	21
		Current state	89	59.73	**30.19**	66	**55**
		Mean difference	87	25.91^***^	–	–	–
20 The necessity of each business trip is weighed up. Everything that can be handled adequately virtually will be done virtually	JAC(2)	D	96	1.97	1.29	2	–
		EP 2030	91	80.74	14.32	80	(18)
		Current state	88	**71.74**	27.3	**80**	34
		Mean difference	81	7.52^**^	–	–	–
27 Remote work offerings are deliberately used by organizations to save costs (e.g., by dissolving rental space, needing less office cleaning, reducing the company car fleet, not needing support for canteen meals or coffee offerings in the office). These savings pay off—despite new investments in technical infrastructures or more mobile phone contracts	JAC(2)	D	88	0.75	1.88	1	–
		EP 2030	87	74.86	19.27	78	22.5
		Current state	85	36.61	24.37	31	44
		Mean difference	80	37.86^***^	–	–	–
31 Working remotely enables an extreme reduction in personal CO_2_ emissions. Through the reduced use of private and public transport and fewer business trips, every person working remotely makes a contribution to environmental protection	JAC(2)	D	95	**2.41**	1.03	**3**	–
		EP 2030	84	74.52	18.99	75	(18.75)
		Current state	78	59.17	27.92	66.5	42.5
		Mean difference	72	14.42^***^	–	–	–
34 Through the regular use of remote work, there is an opportunity to increasingly mix private and work life. The work–life integration of employees is being redefined. Employees can arrange their own working hours and distribute them flexibly throughout the day	JAC(2); JD	D	89	1.97	1.28	2	–
		EP 2030	88	72.88	19.99	75	25
		Current state	80	43.55	25.59	40	46
		Mean difference	76	29.19^***^	–	–	–
33 Regular remote work eliminates the need to commute or travel to work. This makes it easier to balance work and private life. Employees have more energy for private tasks and can better schedule daily recovery times	JAC(2); JD	D	94	2.24	1.11	**3**	–
		EP 2030	87	71.63	20.79	76	(19)
		Current state	82	50.2	23.29	50	35.75
		Mean difference	76	18.90^***^	–	–	–
12 Due to the regular work in the home office, managers have less insight into their employees’ work. As a result, management is results-oriented. This means that a certain amount of time is allocated to a task without the individual work steps and procedures being questioned and checked by the manager. Micro-management is a thing of the past	JAC(1); JAC(2); LS	D	95	1.28	1.91	2	–
		EP 2030	88	71.52	18.91	75	(17.25)
		Current state	81	45.79	25.5	40	40
		Mean difference	77	25.81^***^	–	–	–
10 Individual agreements (e.g., concerning place of work and working hours) are made between employees and the organization. In doing so, each employee’s individual wishes, which may vary between persons and may also change over time for individual employees, are taken into account. Adjustments can be made accordingly	JAC(1); JAC(2); LS	D	97	2.11	1.32	2	–
		EP 2030	91	70.64	22.6	75	(19.5)
		Current state	89	38.7	25.61	33	40
		Mean difference	82	30.46^***^	–	–	–
29 Employees do not have a fixed workplace. Instead, they look for a suitable working environment, depending on the activity: for example, a quiet room for concentrated work or a modern idea room for creative work	JAC(2)	D	88	0.78	1.99	1	–
		EP 2030	91	69.14	23.84	75	25
		Current state	85	30.59	23.03	23	28
		Mean difference	82	37.90^***^	–	–	–
8 Regular use of remote work requires a high degree of self-management skills from employees in order to be able to use the new work situation effectively. The risk of psychological stress in the workplace increases. Employees must shape their work and working conditions themselves and receive appropriate support from the organization in order to successfully master the challenges	JAC(1); JAC(2); SVU; JD	D	89	1.3	1.81	2	–
		EP 2030	89	68.07	20.84	71	(20)
		Current state	89	41.16	27.55	35	43
		Mean difference	83	26.07^***^	–	–	–
6 Social contacts are becoming more relevant due to the isolation in the home office. Offices are evolving from pure workspaces into social meeting spaces and socialization venues for employees	JAC(2); Social	D	95	1.58	1.4	2	–
		EP 2030	83	65.93	22.18	70	(20)
		Current state	84	40.85	28.4	31	47
		Mean difference	76	24.66^***^	–	–	–
11 Regular remote work reduces communication and exchange between employees. As a result, they are increasingly dependent on feedback from others, which is also provided by digital tools	JAC(2); JF; Social; Tech	D	91	−0.18	1.77	0	–
		EP 2030	83	65.25	25.03	71	27
		Current state	81	37.3	27.43	32	48
		Mean difference	75	27.61^***^	–	–	–
25 Work is flexible for employees. Working remotely is not only possible from home but also from anywhere in the world. Many people no longer have just one place of residence and work but several (e.g., in the country and in the city, or even at home and abroad)	JAC(2)	D	88	1.61	1.61	2	–
		EP 2030	85	62.79	25.44	65	30
		Current state	86	29.37	24.68	23.5	30
		Mean difference	80	32.23^***^	–	–	–
5 Virtual meetings are no longer run via video conferences or telephone calls. Advances in digitization and virtualization have made classic live meetings possible in virtual space. Virtual meetings hardly differ from traditional meetings anymore	JAC(2); Tech	D	88	1.32	1.53	1.5	–
		EP 2030	88	61.74	28.25	70	44.25
		Current state	91	16.12	25.25	5	(20)
		Mean difference	84	43.89^***^	–	–	–
2 In order to support employees in remote work in using their individual resources and reducing demands, companies provide employees with analysis options (e.g., in the form of questionnaires). With the evaluation of the questionnaire, the employees receive tips, hints, and support offers tailored to them	JF; JD	D	88	1.18	1.71	2	–
		EP 2030	74	61.64	27.75	68.5	40
		Current state	80	18.55	21.28	10	29
		Mean difference	68	41.90^***^	–	–	–
28 Regular remote work means that new leadership structures are needed. Shared leadership, in which all team members take on certain leadership tasks in individual phases (such as coordinating tasks, setting priorities, showing mutual appreciation, or initiating change), is establishing itself as a new leadership paradigm	JAC(1); JAC(2); SVU; Social; LS	D	87	1.66	1.41	2	–
		EP 2030	80	59.5	23.65	65	26.25
		Current state	87	26.31	21.84	20	29
		Mean difference	77	32.23^***^	–	–	–
19 Regular remote work means that employees are much less tied to the organization. As a result, they change organizations more frequently and more quickly	JAC(2)	D	83	−1.59	1.33	−2	–
		EP 2030	84	57.96	23.41	63.5	35
		Current state	78	27.36	20.76	24.5	30
		Mean difference	72	27.59^***^	–	–	–
3 Working from a home office means that tasks need to be more clearly defined, formulated, and mapped out. This makes it easy to relocate work packages worldwide and creates an openness to outsourcing individual work orders. Work packages are outsourced worldwide via platforms. These individual tasks, which are often temporary, are taken on by contractors from all over the world or mobile, independent “working nomads.”	JAC(1); JAC(2); SVU	D	86	−0.93	1.88	−2	–
		EP 2030	84	57.82	25.83	65	36.25
		Current state	84	26.74	21.44	23.5	29.25
		Mean difference	79	30.60^***^	–	–	–
32 Employees have the impression that they have to be available all the time. The assumption of constant availability makes switching off almost impossible and leads to stress	JAC(2); JD	D	95	−2.43	1	−3	–
		EP 2030	87	56.99	25.63	62	36.5
		Current state	81	54.31	25.63	60	45
		Mean difference	77	4.17	–	–	–
1 Employees seek out off-site co-working spaces close to home where they can work undisturbed while making valuable social and work-related connections with people outside their own organizations	JAC(2); Social	D	84	1.08	1.78	1.5	–
		EP 2030	83	56.61	25.59	60	40
		Current state	87	16.91	15.84	12	(16.5)
		Mean difference	79	39.03^***^	–	–	–
22 Virtual meetings are supported by artificial intelligence, which, for example, takes over the structuring and logging of the meeting and automatically provides suitable additional information as needed, depending on the statements of the participants	Social; Tech	D	88	0.91	1.75	1	–
		EP 2030	84	55.73	26.74	60	45.75
		Current state	88	6.1	9.9	0	(10)
		Mean difference	81	48.46^***^	–	–	–
17 Due to regular remote work, employees have a higher risk of working more than they should. Excessive working, work intensification, and the resulting consequences for health (e.g., burnout) are a major problem	JAC(2); JD	D	93	−2.54	0.94	−3	–
		EP 2030	81	55.56	25.9	62	40
		Current state	82	53.43	25.43	60	38
		Mean difference	74	1.69	–	–	–
24 In order to stay connected with colleagues working virtually, organizations use virtual tunnels for informal communication. In certain rooms (e.g., the kitchen or break rooms), there are on-site screens on which colleagues working remotely can be connected in order to promote spontaneous and informal exchange between all employees	Social; Tech	D	91	0.6	**2.07**	1	–
		EP 2030	86	54.21	29.89	65	50.25
		Current state	91	15.04	20.23	7	22.5
		Mean difference	83	37.74^***^	–	–	–
30 Regular remote work reduces communication and exchange between employees on the same team. There is a shared understanding (shared mental models) about competencies within the team, tasks (goals), times (deadlines, processing times), and the shared use of technologies. This shared understanding enables high performance	JAC(1); JAC(2); Social	D	83	−0.2	2.05	−1	–
		EP 2030	77	54.18	26.63	60	40
		Current state	76	34.41	24.1	30	42
		Mean difference	70	18.59^***^	–	–	–
23 Regular remote work puts a strain on employees’ social structures. More effort has to be made to stay in contact with colleagues and to build collegial relationships with each other. The working atmosphere in organizations often seems cool, and communication has a strong focus on work-related topics	JAC(2); Social; JD	D	95	−1.86	1.43	−2	–
		EP 2030	84	53.42	25.6	60	45
		Current state	85	49.91	25.08	59	40
		Mean difference	79	3.95	–	–	–
15 Employees who regularly work remotely are more focused and efficient. This results in higher productivity, which supports the introduction of the 4‑day week	JAC(2)	D	91	1.54	1.75	2	–
		EP 2030	81	50.84	29.09	50	47
		Current state	85	21.89	21.85	15	25
		Mean difference	74	28.55^***^	–	–	–
13 Regular remote work increasingly leads to individual overload, which affects both work and private life. People who live with others (e.g., couples, families, shared apartments) are easily irritable. People who live alone become lonely	JAC(2); Social; JD	D	94	−2.68	0.59	−3	–
		EP 2030	86	50.34	25	55.5	40.75
		Current state	86	57.34	25.05	63.5	44
		Mean difference	79	−5.96	–	–	–
35 Regular remote work results in the loss of professional networks within the organization. This has a negative impact on the sharing of knowledge within organizations and learning from each other, as well as on the career opportunities of individuals	SVU; JF; Social	D	93	−2.35	1.01	−3	–
		EP 2030	85	46.07	24.99	49	45
		Current state	83	44.94	28.46	40	50
		Mean difference	76	1.09	–	–	–
9 In organizations, those who play a major role in decision-making will be found on site. Those who do more of the legwork will work remotely	JAC(1); JAC(2); LS	D	79	−1.47	1.53	−2	–
		EP 2030	84	45.93	27.48	42.5	45.25
		Current state	83	41.08	29.63	33	54.5
		Mean difference	77	6.05^*^	–	–	–
14 Working remotely becomes a status symbol and motivational element. Employees are rewarded for their good work by the company granting them workdays at home	LS	D	88	−1.5	1.94	−2	–
		EP 2030	79	41.43	**30.77**	34	**54.5**
		Current state	76	25.79	25.5	20	36.25
		Mean difference	71	12.71^***^	–	–	–
21 The performance evaluation of employees is done by machine. Not only is the quantity of work affected, but the quality of the work is also evaluated automatically via an algorithm without a human being involved	JF; LS; Tech	D	93	−2.19	1.38	−3	–
		EP 2030	86	40.07	26.54	32	40
		Current state	87	12	15.8	5	(18.5)
		Mean difference	82	27.47^***^	–	–	–
4 Virtuality in meetings makes overarching strategic exchange within organizations more difficult. Far-reaching agreements on complex issues and strategic decisions cannot be reached in virtual meetings. Viable solutions fail to materialize	JAC(1)	D	90	−2.23	1.23	−3	–
		EP 2030	81	35.51	27.77	27	42
		Current state	86	37.21	27.74	28	43
		Mean difference	77	0.28	–	–	–
18 Regular remote work reduces involvement in the work. Employees are less engaged in their work, rarely show any willingness to help, and hardly ever bring new ideas into their work	JAC(2); SVU; Social	D	91	−2.62	0.73	−3	–
		EP 2030	82	32.39	24.09	25	32.5
		Current state	82	27.48	21.92	20	26.5
		Mean difference	76	4.47	–	–	–
7 Instead of working, employees exploit remote work for private purposes. During working hours, they meet with private contacts, occupy themselves with their own property (e.g., house and garden work), or pursue private sideline activities	JAC(2)	D	90	−1.96	1.51	−3	–
		EP 2030	76	27.46	21.74	21.5	21.75
		Current state	80	26.24	22.64	20	21
		Mean difference	71	0.78	–	–	–
16 Digital monitoring programs are experiencing brisk sales. Videos of an employee’s screen are recorded at regular intervals. Every 10 min, the webcam also snaps a photo to ensure that employees are available at their workstations	JAC(1); LS; Tech	D	97	−2.9	0.42	−3	–
		EP 2030	84	27.15	24.05	20	25.25
		Current state	89	8.19	10.28	4	(12)
		Mean difference	81	18.33^***^	–	–	–

Brackets indicate consensus (IQR ≤ 20); bold numbers indicate max values for D, EP, current occasion, and IQR; descended sorting by estimated probability in 2030

*n* sample size, *JAC(1)* Job Autonomy and Control (decision-making as part of work processes), *JAC(2)* Job Autonomy and Control (choice over where and when to work), *SVU* Skill variety and use, *JF* Job feedback and related, *Social* Social and relational, *JD* Job demands, *LS* Leadership, *Tech* Technology, *D* desirability, *EP* estimated probability, *Mean difference* difference between EP 2030 and current state

*p ≤ 0.05, **p ≤ 01, ***p ≤ 0.001

### Desirability and probability of current and future scenarios

Our second research question dealt with the desirability of the derived scenarios. Similarly, RQ3 was concerned with the estimated probabilities of the scenarios at the present time and in the future. To serve this purpose, we conducted a quantitative Delphi survey and calculated descriptive statistics. Table 1 presents the results for desirability and estimated probability, as well as the estimated current level of expression, sorted by estimated probability for 2030. In addition, the interquartile range (IQR) indicates the degree of agreement between the experts interviewed, denoting the absolute difference between the third and the first quadrant and, thus, comprising the middle 50% of all responses. Smaller IQR values indicate a greater consensus among respondents (von der Gracht 2012; Rayens and Hahn 2000; Scheibe et al. 2002). Consensus can be assumed for those scenarios in which the IQR is no more than 2 units on a 10-point scale (Scheibe et al. 2002) or a 100-point scale (0–100%); in other words, IQR ≤ 20 (Bokrantz et al. 2017).

The average desirability was rated as *M*_D_ = −0.04 (*SD* = 1.45) on average. This result indicates that the scenarios were mostly rated as rather neutral (a mean value of 0 would indicate a neutral evaluation of the scenarios). Nonetheless, the standard deviation reveals a notable variation regarding desirability ratings among the experts. The current likelihood of the scenarios was rated as *M*_CS_ = 35.20 (*SD* = 23.61), while the estimated probability for 2030 was evaluated as *M*_EP_ = 56.60 (*SD* = 24.12). A paired *t* test for mean differences, *t* (102) = 16.238, *p* < 0.001, for a calculated *M*_diff_ = 22.19 indicates a significant increase in estimated probability from 2021 to 2030. The t‑tests were assessed according to the requirements and corrected accordingly. In 2021, six scenarios were rated with a probability above 50%. This number increased to 25 scenarios in 2030.

In all, these results indicate mixed opinions among experts regarding the desirability of the chosen scenarios. In reference to the probabilities, the standard deviations for all scenarios were quite large, on average, for the present and future. Nevertheless, the significant increase in estimated probabilities should be highlighted, as it shows an optimistic outlook for the future.

### The connection between desirability and probability in the present and future

Our fourth research question dealt with the connection between desirability and probability. To determine whether the two variables converged over time, we calculated correlations between desirability and estimated probabilities. The relationship between desirability and current probability can be classified as a medium-strong effect, *r* = 0.33 (*p* < 0.001). In contrast, the relationship between desirability and future probability indicates a stronger effect, *r* = 0.43 (*p* < 0.001). This result implies that the connection between desirability and probability grows stronger over time. This trend was found for all scenarios. In all, the growing link between desirability and likelihood adds to previous results highlighting the experts’ optimistic outlook for the future.

For a deeper analysis of the connection between desirability and probability, we created scatterplots for current and future scenarios. To identify overall trends, we divided each scatterplot into quadrants. The top left quadrant indicates scenarios that were rated as highly desirable yet unrealistic, while the bottom left quadrant indicates scenarios that were rated as less desirable yet also unrealistic. In comparison, the top right quadrant indicates scenarios that were rated as highly desirable and realistic, while the bottom right quadrant indicates scenarios that were less desirable yet realistic.

**Fig. 2:**
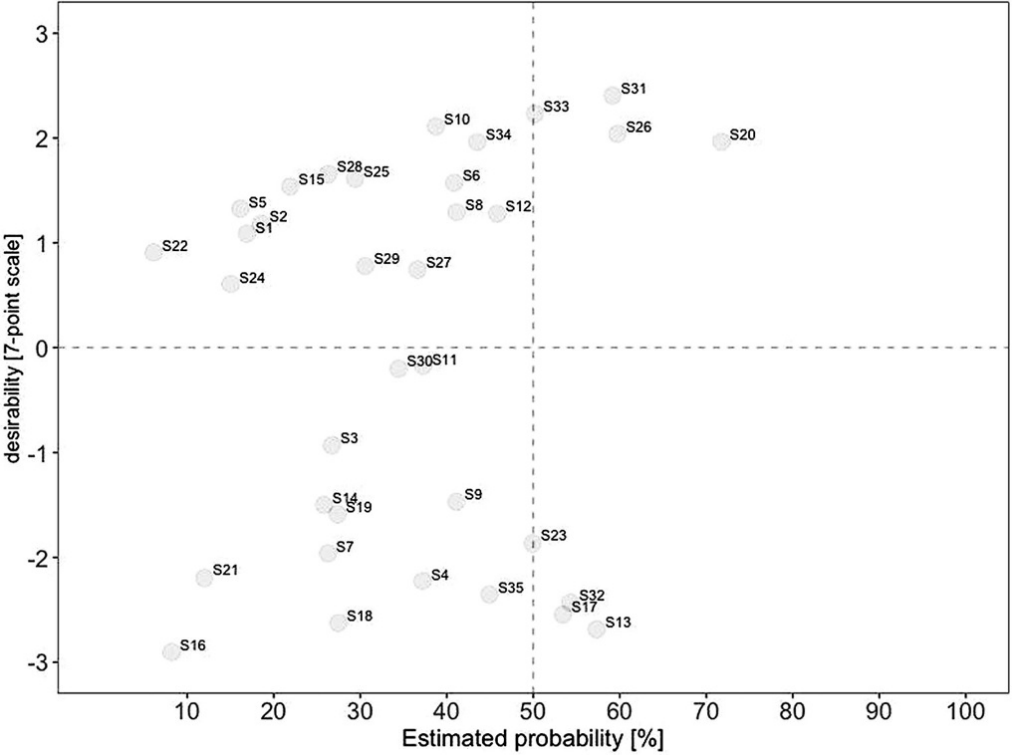
Desirability–Probability Scatterplot of Scenarios for 2021. (*Top left quadrant* = high desirability/low probability; *bottom left quadrant* = low desirability/low probability; *top right quadrant* = high desirability/high probability; *bottom right quadrant* = low desirability/high probability)

Fig. 2 displays the estimated probabilities for 2021, plotted on the x‑axis against the respective estimated desirability on the y‑axis. Notably, the probability is less dispersed for scenarios with low desirability than for scenarios with higher desirability. Overall, only six scenarios were estimated as realistic (i.e., with an estimated probability of notably over 50% for 2021). Three of these scenarios were rated as highly desirable (i.e., a desirability score of notably over 0). These included (#31) working remotely contributes to environmental protection, (#26) increased use of hybrid meetings so that everyone can participate regardless of their geographical position, and (#20) stricter examination of business trips regarding necessity. In contrast, three scenarios were rated as highly realistic yet undesirable. These included (#17) higher risk for employees to work more than they should, (#32) constant availability felt by employees, and (#13) remote work increasingly leads to individual overload.

**Fig. 3:**
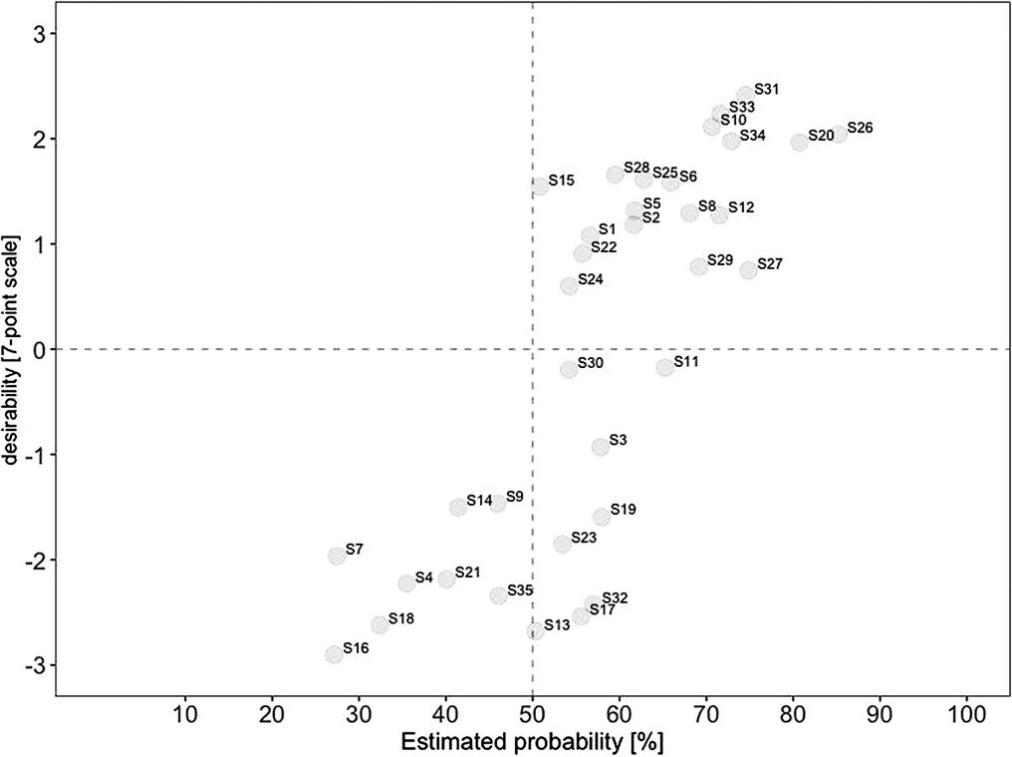
Desirability–Probability Scatterplot of Scenarios for 2030. (*Top left quadrant* = high desirability/low probability; *bottom left quadrant* = low desirability/low probability; *top right quadrant* = high desirability/high probability; *bottom right quadrant* = low desirability/high probability)

Similarly, Fig. 3 illustrates the estimated probability for 2030 on the x‑axis against the respective estimated desirability on the y‑axis. Concerning desirability, (#31) working remotely contributes to environmental protection retained the highest score. At the same time, (#26) increased use of hybrid meetings so that everyone can participate regardless of their geographical position was rated as the most realistic-and-desirable scenario. In comparison, (#17) higher risk for employees to work more than they should was rated as the most undesirable among the realistic scenarios. We also found a significant increase in probability by over 40% for three scenarios, including (#22) virtual meetings supported by artificial intelligence, (#5) meetings take place in virtual space thanks to advances in digitization and virtualization, and (#2) companies provide employees with analysis options (e.g., in the form of questionnaires). As a result, these scenarios shifted from highly desirable yet unrealistic to desirable-and-likely. In contrast, only one scenario decreased in estimated probability, with a mean difference of −5.96: (#13) remote work increasingly leads to individual overload.

### Work design categories relevant to the future of work

RQ5 and RQ6 asked about the relevance of work design characteristics for the future of work. In this context, we adapted and extended the five work design categories from Parker and Grote (2020). In summary, a total of 27 scenarios accounted for aspects of *job control*, 11 for *social and relational* aspects, nine for *job autonomy*, eight for *job demands*, seven each for *leadership* and *technology*, five for *skill variety and use*, and 4 for *job feedback*. We found that the most scenarios were related to *task characteristics* (29), followed by* knowledge characteristics* (12),* social characteristics* (11), and* contextual characteristics *(eight). Due to the large number of scenarios that fit into the extended categories, we can state that work characteristics play a significant role in designing a successful future of work.

For a deeper analysis of the work design characteristics, we examined the connection between desirability and probability in the present and future for each category. Consistent with our previous analyses, we created scatterplots for every category. In comparison to the overall scatterplots, each categorical scatterplot contains probability ratings for both 2021 and 2030, depicted by different symbols.

#### Task characteristics

The large number of assigned scenarios shows the relevance of job autonomy and control aspects. A bigger focus on results instead of micro-management and more individual agreements between employees and organizations were viewed as crucial resources of future work. In contrast, individual overload and pressure of constant availability represented undesirable aspects of WFH, even though they are already realistic parts of everyday work life. In comparison, aspects of job feedback were rated as less relevant. The development of advanced analysis options for tips, hints, and support was viewed as rather desirable, whereas the anticipated reduction of communication and exchange between employees was seen as rather undesirable. The following figures and their associated descriptions offer details about the results for each of the three categories.

##### Job control

**Fig. 4:**
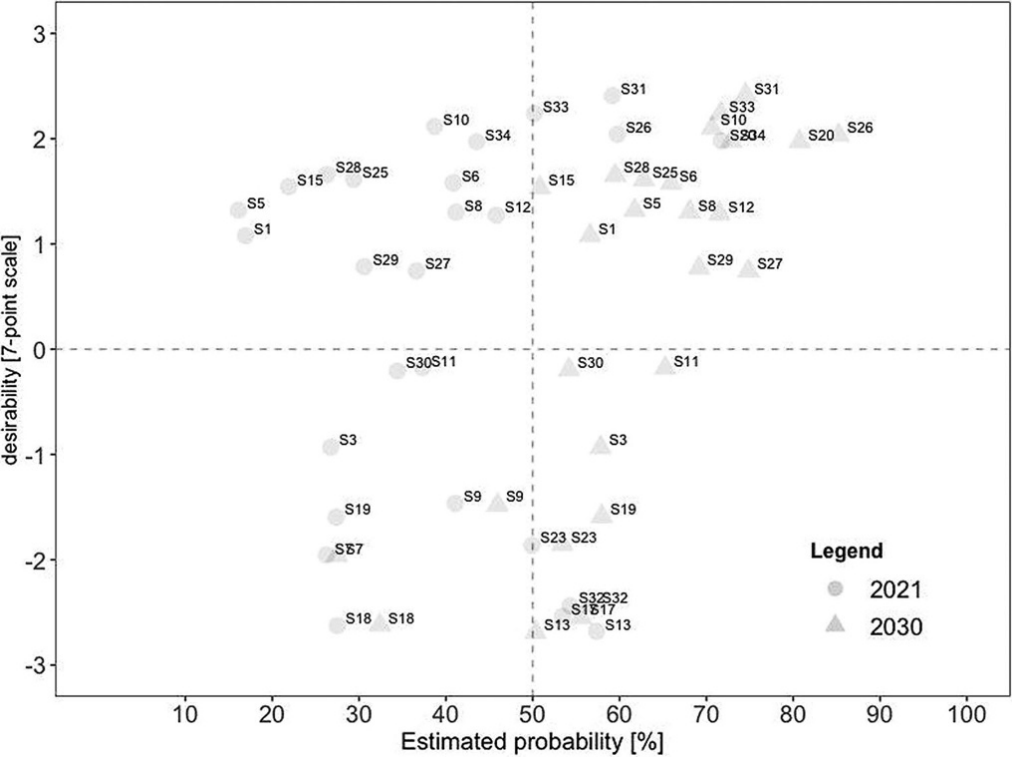
Desirability–Probability Scatterplot of Scenarios Containing Aspects of Job Control (Choice Over Where and When to Work). (*n* = 27 scenarios; *top left quadrant* = high desirability/low probability; *bottom left quadrant* = low desirability/low probability; *top right quadrant* = high desirability/high probability; *bottom right quadrant* = low desirability/high probability; *triangle* = 2030; *circle* = 2021)

Scenarios promoting aspects of *job control* are displayed in Fig. 4. In this area, 15 of the 27 scenarios were rated as highly desirable and realistic, including (#31) working remotely contributes to environmental protection and (#26) increased use of hybrid meetings so that everyone can participate regardless of their geographical location. In contrast, eight were estimated to be rather undesirable-yet-realistic. In 2021, the most undesirable was (#13) remote work increasingly leads to individual overload. In 2030, the choice was (#17) higher risk for employees to work more than they should. Notably, the mentioned scenarios were already seen as realistic in 2021 and as remaining viable in the future.

##### Job autonomy

**Fig. 5:**
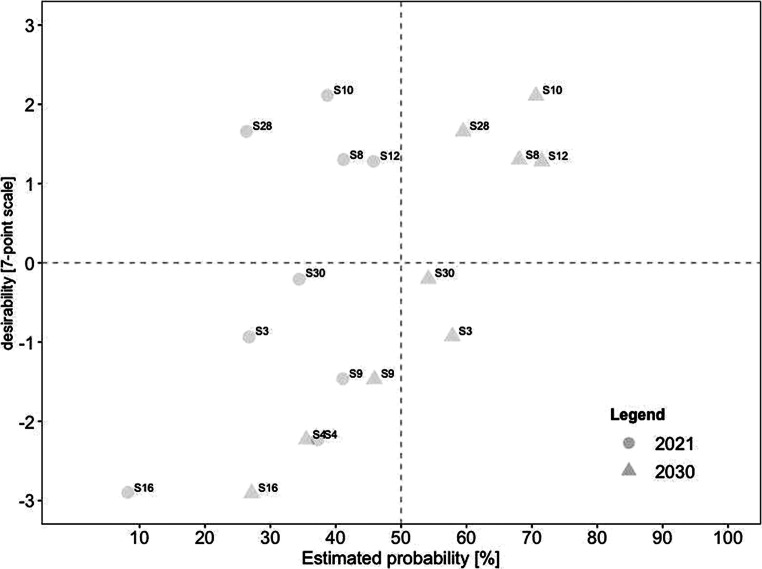
Desirability–Probability Scatterplot of Scenarios Containing Aspects of Job Autonomy (Decision-Making as Part of Work Processes). (*n* = 9 scenarios; *top left quadrant* = high desirability/low probability; *bottom left quadrant* = low desirability/low probability; *top right quadrant* = high desirability/high probability; *bottom right quadrant* = low desirability/high probability; *triangle* = 2030; *circle* = 2021)

Fig. 5 displays the nine scenarios promoting aspects of *job autonomy*. None of the scenarios was estimated as realistic in 2021, whereas six became highly likely in 2030. Three scenarios were seen as desirable and realistic in the future, including (#10) individual agreements (e.g., on place of work and working hours) are made between employees and the organization, (#8) high degree of self-management skills from employees as requirement for remote work, and (#12) management becomes more results-oriented and less micro-management-oriented. In contrast, (#3) outsourcing individual work orders by working remotely was rated as realistic yet undesirable in 2030.

##### Job feedback

**Fig. 6:**
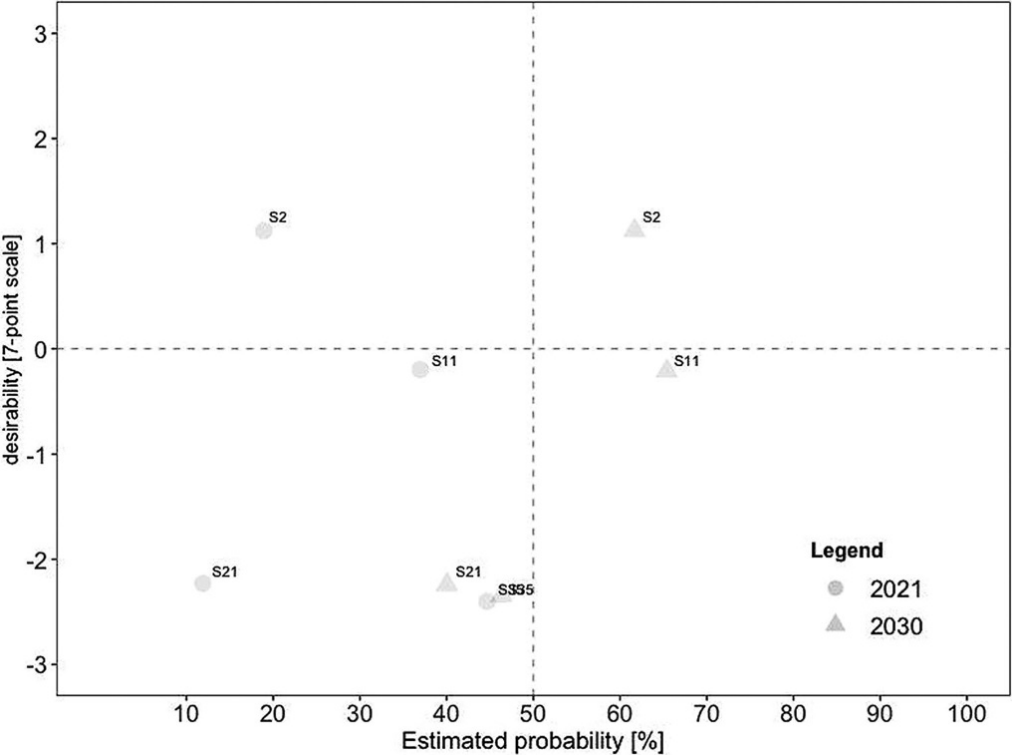
Desirability–Probability Scatterplot of Scenarios Containing Aspects of Job Feedback and Related. (*n* = 4 scenarios; *top left quadrant* = high desirability/low probability; *bottom left quadrant* = low desirability/low probability; *top right quadrant* = high desirability/high probability; *bottom right quadrant* = low desirability/high probability; *triangle* = 2030; *circle* = 2021)

Only four scenarios promoted aspects of job feedback. Not surprisingly, it was the category mentioned least by our experts. The scenarios are illustrated in Fig. 6. None of the scenarios were rated as realistic in 2021, with two becoming viable in the future. While (#2) companies provide employees with analysis options (e.g., in the form of questionnaires) was rated as rather undesirable, (#11) regular remote work reduces communication and exchange between employees was rated as moderately undesirable.

#### Knowledge characteristics

Leadership was seen as crucial resources of work. In comparison, we found a negative scenario related to* skill variety and use *as a critical aspect of future work.

##### Leadership

**Fig. 7:**
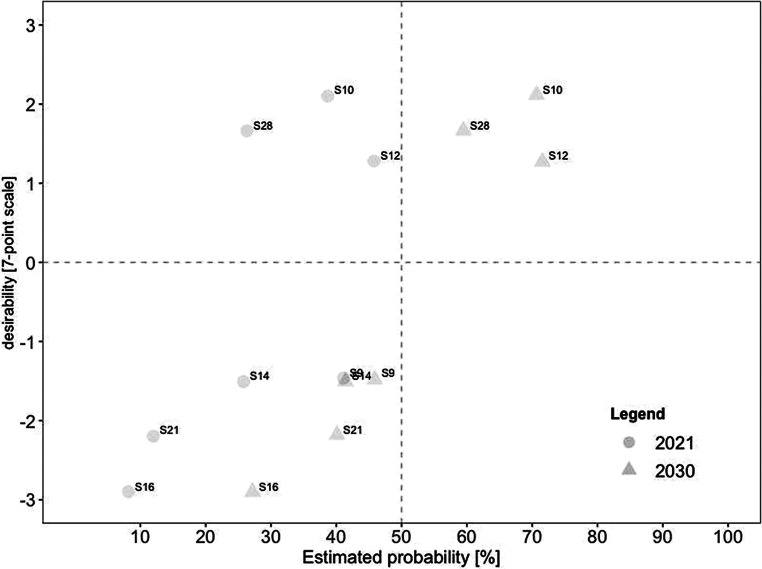
Desirability–Probability Scatterplot of Scenarios Containing Aspects of Leadership. (*n* = 7 scenarios; *top left quadrant* = high desirability/low probability; *bottom left quadrant* = low desirability/low probability; *top right quadrant* = high desirability/high probability; *bottom right quadrant* = low desirability/high probability; *triangle* = 2030; *circle* = 2021)

The seven scenarios promoting aspects of leadership are displayed in Fig. 7. The figure shows a large gap between the three desirable and four undesirable scenarios. None were rated as currently realistic, and only the three desirable scenarios were expected to become realistic in the future. These included (#10) individual agreements (e.g., on place of work and working hours) are made between employees and the organization, (#28) regular remote work requires new leadership such as shared leadership, and (#12) management becomes more results-oriented and less micro-management-oriented.

##### Skill variety and use

**Fig. 8:**
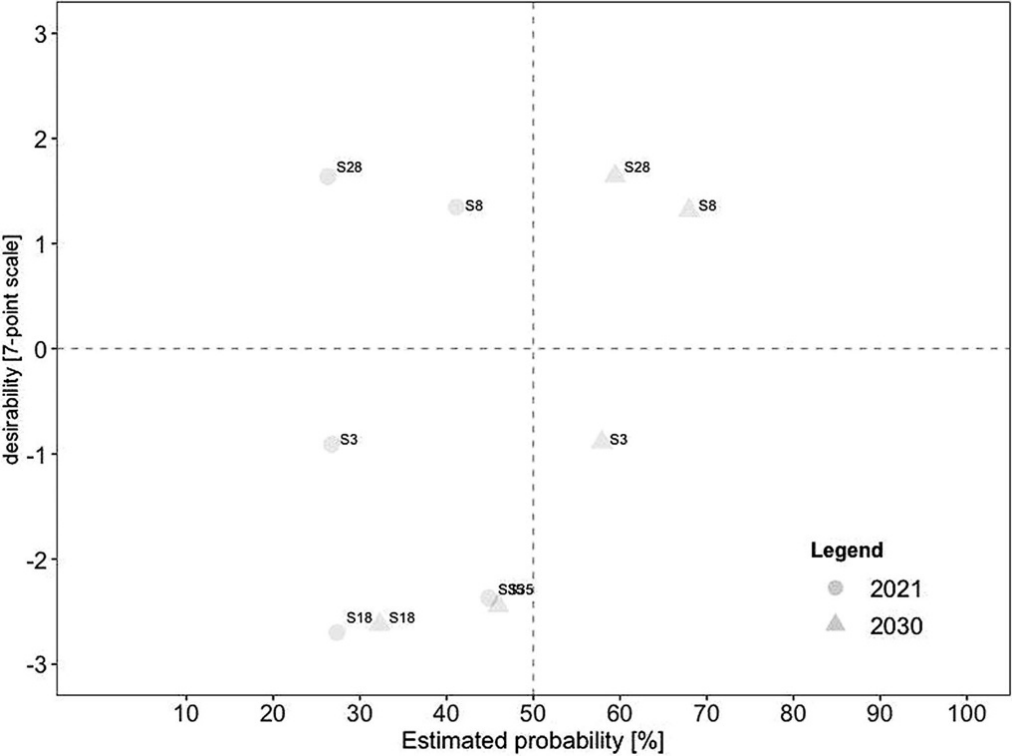
Desirability–Probability Scatterplot of Scenarios Containing Aspects of Skill Variety and Use. (*n* = 5 scenarios; *top left quadrant* = high desirability/low probability; *bottom left quadrant* = low desirability/low probability; *top right quadrant* = high desirability/high probability; *bottom right quadrant* = low desirability/high probability; *triangle* = 2030; *circle* = 2021)

Scenarios promoting aspects of *skill variety and use *are represented in Fig. 8. None of the five scenarios were rated as realistic for 2021, with three becoming viable in 2030. Two of those were rated as highly desirable, including (#28) regular remote work requires new leadership (such as shared leadership) and (#8) high degree of self-management skills from employees as requirement for remote work. In contrast, (#3) outsourcing individual work orders by working remotely was rated as rather undesirable-yet-realistic in the future.

#### Social characteristics

##### Social and relational

**Fig. 9:**
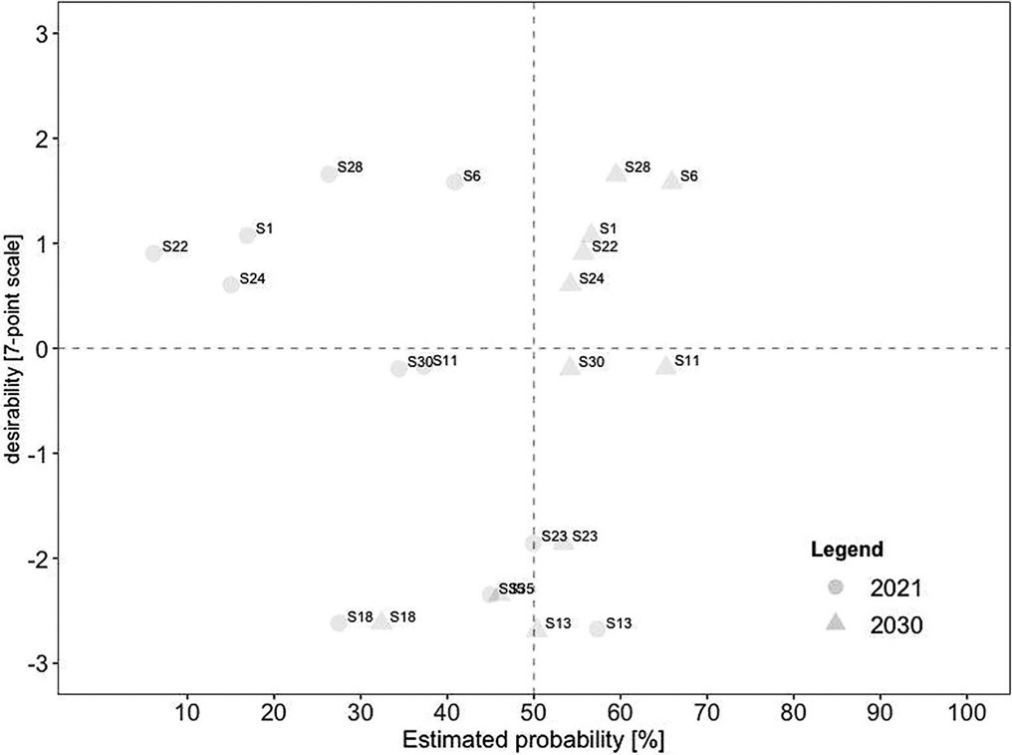
Desirability–Probability Scatterplot of Scenarios Containing Social and Relational Aspects. (*n* = 11 scenarios; *top left quadrant* = high desirability/low probability; *bottom left quadrant* = low desirability/low probability; *top right quadrant* = high desirability/high probability; *bottom right quadrant* = low desirability/high probability; *triangle* = 2030; *circle* = 2021)

This category, which featured in 11 scenarios, was seen as the second most important work characteristic. Fig. 9 demonstrates the social and relational aspects of the scenarios. Notably, only one scenario was realistic in 2021, and it was also the most undesirable: (#13) remote work increasingly leads to individual overload. Also noteworthy is that scenario #13 was expected to become unlikely in 2030. Two scenarios were rated desirable and realistic in 2030, including (#28) regular remote work requires new leadership, such as shared leadership, and (#6) offices evolving from pure workspaces into social meeting spaces and socialization venues for employees.

#### Contextual characteristics

##### Job demands

**Fig. 10:**
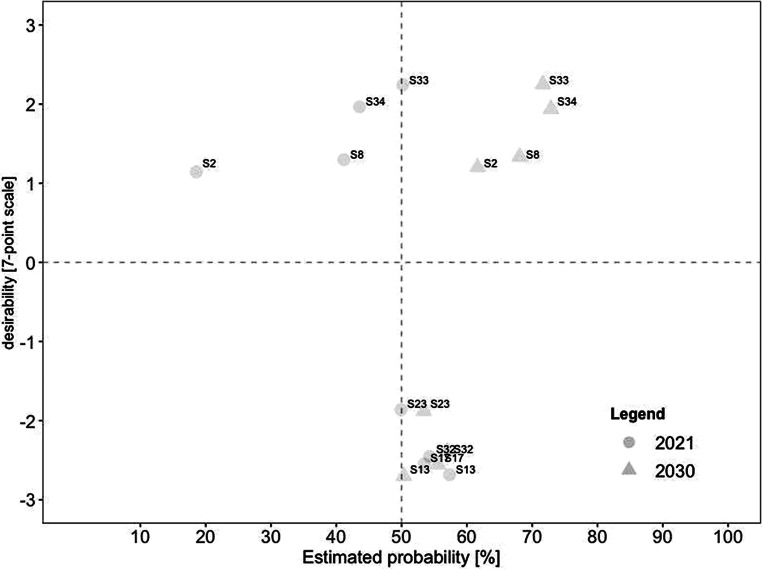
Desirability–Probability Scatterplot of Scenarios Containing Aspects of Job Demands. (*n* = 8 scenarios; *top left quadrant* = high desirability/low probability; *bottom left quadrant* = low desirability/low probability; *top right quadrant* = high desirability/high probability; *bottom right quadrant* = low desirability/high probability; *triangle* = 2030; *circle* = 2021)

Contextual characteristics exhibited a large discrepancy between highly desirable and undesirable, as can be seen in Fig. 10. The eight scenarios were equally split between desirable and undesirable. Notably, none of the four desirable scenarios related to job demands were seen as currently realistic, even though all were expected to become viable in 2030. In contrast, all four undesirable scenarios were rated as already realistic in 2021. The two scenarios rated as highly desirable and realistic in the future were (#33) regular remote work eliminates the need to commute or travel to work and therefore makes it easier to balance work and private life and (#34) the work–life integration of employees is being redefined and offers an opportunity to increasingly mix private and work life. Highly undesirable scenarios included (#32) constant availability felt by employees, (#17) higher risk for employees to work more than they should, and again (#13) remote work increasingly leads to individual overload.

#### Technology

**Fig. 11:**
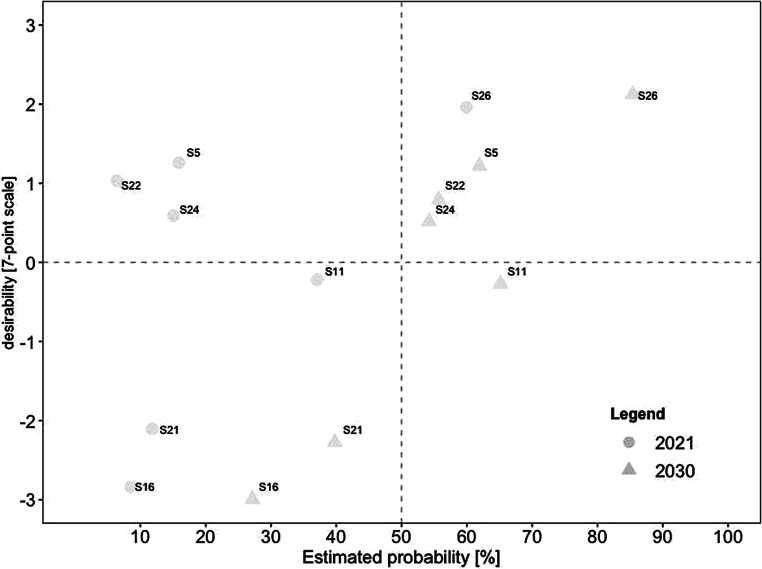
Desirability–Probability Scatterplot of Scenarios Containing Aspects of Technology. (*n* = 7 scenarios; *top left quadrant* = high desirability/low probability; *bottom left quadrant* = low desirability/low probability; *top right quadrant* = high desirability/high probability; *bottom right quadrant* = low desirability/high probability; *triangle* = 2030; *circle* = 2021)

The seven scenarios related to technology are displayed in Fig. 11. Only one of the scenarios was rated as currently realistic: (#26) increased use of hybrid meetings so that everyone can participate regardless of their geographical position. This item was also the most desirable scenario. Three more scenarios were expected to become viable in the future, including (#5) meetings take place in virtual space thanks to advances in digitization and virtualization, (#22) virtual meetings are supported by artificial intelligence, and (#24) organizations use virtual tunnels for informal communication.

## Discussion

In this Delphi-based study, we aimed to determine future work developments in a post-COVID-19 world. Overall, the predictions that emerged are optimistic, as the future scenarios describe more benefits than detriments, with a significant number of desirable scenarios estimated to become realistic in 2030. Advantages are mainly seen on the organizational and individual levels, whereas risks are perceived as affecting at the team level.

On the organizational side, technology and leadership are the most critical factors in the context of remote and mobile work. Advancements in ICT, such as virtual tunnels and the use of artificial intelligence, are seen as crucial developments for the future. The focus on technologies is consistent with research predicting that new technologies will create significant changes for work, such as increased human–robot interactions and the use of smart technologies (Brynjolfsson et al. 2018; Huang and Rust 2018). In our study, advancements in ICT that enhance work flexibility and job feedback are revealed as particularly important job resources for the future. These resources are also highlighted by the desirable leadership behaviors that are seen as an integral part of future work. Therefore, leadership styles should focus on result orientation over micro-management and an increase in employee participation via shared leadership. These findings are in line with current leadership research that has postulated a growing need for relation-oriented leadership behaviors and empowering leaders. In the context of the digital transformation, leadership should focus on sharing responsibilities and granting autonomy, which facilitates intrinsic motivation, shared mental models and promotes feelings of vitality (Ali et al. 2018; Kleine et al. 2019; Lungeanu et al. 2022; Mertens and Recker 2020).

For employees, the increase in remote work is viewed as a great opportunity to improve work–life balance. In this regard, advances in feedback options (such as analysis tools and questionnaires) and more personal responsibility for task organization are seen as important tools to ensure effective work–life integration. These findings are in line with a recent meta-analysis that showed organizational and leadership support to be crucial for employees to thrive at work (Kleine et al. 2019). Accordingly, employees wish for a work environment that conveys a sense of security and places high value on employees’ well-being. These conditions promote work motivation and job satisfaction, leading to less fatigue and burnout (e.g., Berg et al. 2013; Riaz et al. 2018; Taneva and Arnold 2018).

Regarding teamwork, organizations are concerned about the social and relational aspects of future work. Remote and mobile work are growing significantly, leading to expectations that social ties among employees will require more effort to build and maintain. This development is seen as a challenge, hampering team cohesion and decreasing organizational commitment. The results are consistent with studies examining the importance of social support in the work context, which showed social support by colleagues (e.g., by providing help or sharing information) as a relevant job resource (Bakker and Demerouti 2007; Hobfoll 1989; Morgeson et al. 2013). In ad-hoc teams, the lack of personal knowledge and mental models is seen as a risk for lower team cohesion (Bushe and Chu 2011). For virtual teamwork, the importance of team building has been stressed, as the promotion of collaborative interactions and establishment of a supportive team climate are critical requirements for virtual teams to be successful (Battilana et al. 2010; Liao 2017). While the weakening of team boundaries and strengthening of cross-divisional cooperation indicates a negative influence on teamwork, this phenomenon could also be seen as an opportunity for organizations. Building on social network research, a large number of weak ties represents an advantage compared to a small number of strong ties as found in permanent team membership. This network pattern provides access to new and important information while the position acts as a bridge between different communities (Granovetter 1973; Granovetter and Soong 1983; Lin et al. 2020). Wu et al. (2021) were also able to show the relevance of networks in general and the impact of different network behaviors on performance in times of crisis (e.g., the COVID-19 pandemic).

Beyond future developments, several scenarios stand out that are already seen as realistic in the present. The desire for a reduction of emissions and a stricter examination of business travel is particularly strong, which fits the current socio-political climate and illustrates its growing relevance (Kuzemko et al. 2020; Wells et al. 2020). Workplace flexibility is already seen as a realistic key for organizations to deal with the current changes caused by digitalization. In particular, location-independent work via virtual or hybrid meetings is viewed as a critical measure that can be implemented straight away to remain competitive while ensuring employees’ satisfaction and productivity. This assumption is consistent with research showing a growing trend in terms of redesigning organizational structures via increasing use of cross-location teams, new opportunities for employees to move beyond company boundaries, and flexible network patterns replacing rigid hierarchies (Boudreau et al. 2015; Parker and Grote 2020; Zammuto et al. 2007). In contrast, work scenarios that are perceived as currently realistic yet undesirable relate primarily to aspects of the workload. At this point in time, the risk of overload and constant availability already exists and is seen as highly problematic. Employees’ difficulties in coping with rapid technology changes have been well established in scientific literature. Brod coined the term Technostress as early as 1984 to describe the pressure and stress caused by individuals’ inability to adapt to new technologies in a healthy way (Brod 1984). Technostress has been found to have a negative impact on performance, with excessive workload and insufficient skills as the two major contributing factors (Suharti and Susanto 2014). This negative effect is strengthened by employees’ fears that companies will increasingly outsource work due to a growing number of people working as freelancers. Researchers have expressed agreement that the workforce structure will change significantly, especially for less skilled workers (Brynjolfsson et al. 2018; Dellot and Wallace-Stephens 2017; Huang and Rust 2018). As a result, uncertainty and job insecurity due to the unpredictable consequences of digitalization have been found to cause anxiety in employees (Pfaffinger et al. 2020). These results correspond with the anticipated positive developments we found in our study. Advancements in technologies and better feedback options are seen as “glimmers of hope”, as they are expected to improve work–life balance and, thus, serve as buffers against individual overload and work-related stress.

### Implications

Our study contributes to the research and practice focusing on the future of work that has gone through dramatic changes during the COVID-19 pandemic. Deriving and evaluating future scenarios via the Delphi method helps in identifying opportunities and risks in the context of increasing mobile work and virtual collaboration. Our results reveals the complexity of the topic and the wide variety of factors that are involved. For this reason, we integrated the study’s scenarios in work design theory, using it as a framework to increase clarity and comprehensibility. Based on the literature, we adapted work design categories from Parker and Grote (2020) and suggested adding a sub-category related to knowledge characteristics, namely *leadership*. We also examined *technology* as a critical category affecting key work characteristics. Our findings confirm the relevance of the additional categories, as seven scenarios account for aspects of *leadership* and *technology* each. Based on the scenarios related to *task, knowledge*, and *social* and *contextual characteristics* of work design, it is possible to determine which work conditions are desirable and how to achieve them in practice.

We found *task characteristics *to have a crucial influence on the future success of mobile work. Autonomy in decision-making and more feedback options are considered crucial job resources to help cope with the demands of work in the future. In particular, less micro-management and more individual arrangements are important job resources. In contrast, undesirable aspects of *job control* are already part of everyday work life, especially individual overload and the pressure of constant availability. Consequently, organizations must provide specific ICT that promote autonomy and support via feedback and analysis tools. Supplying direct feedback might increase decision-making autonomy and skill variety. At the same time, organizations need to disclose their internal use of digital technologies regarding the monitoring of employees to reduce stress due to feelings of being controlled (Leonardi 2021). In addition, expectations regarding working hours should be clarified. A certain amount of regulation is needed to give mobile work distinct boundaries. Regulation provides structure, thereby reducing the pressure of constant availability (Pfaffinger et al. 2020).

We found* knowledge characteristics *to be equally important as *leadership* and *skill variety* in terms of critical aspects. Regarding *leadership*, participation and allocation of responsibilities are integral resources that promote employees’ motivation and job satisfaction. Therefore, managers must adapt their leadership style to meet the demands of virtual teamwork. In this regard, shared leadership is a promising leadership approach. For a successful implementation, however, managers need relevant knowledge and organizational support. At the same time, we found a negative scenario related to* skill variety and use *that must be addressed. Outsourcing is perceived as a significant future threat because it promotes “working nomads” and freelance work (Glavin et al. 2021). In this regard, it is fundamental for organizations to satisfy their employees’ learning needs. Providing training and workshops to build up ICT skills could be beneficial to help workers regain a sense of control and reduce their feelings of uncertainty.

#### Social characteristics

revolve around the risks we found for teamwork. Undesirable future scenarios describe employees’ concerns about decreasing social contact and collegial relationships. In the same vein, desirable future scenarios highlight the importance of social meeting spaces and socialization venues. As a result, organizations must provide ways to facilitate social exchange among workers. This function can be achieved via redesigning offices and providing attractive work environments. Nevertheless, it is essential for managers to be aware of the relevance of team building and social support for virtual teams (Mütze-Niewöhner et al. 2021).

#### Contextual characteristics

reaffirm our findings regarding work flexibility. Workload and constant availability are seen as current *job demands *(e.g., Schulte et al. 2021). In contrast, mobile work is perceived as a future resource that provides opportunities to improve work–life integration. For mobile work to function as a buffer against job demands, however, ergonomics must be considered. Companies should therefore pay particular attention to user-friendly designs when introducing smart technologies.

Our reasoning regarding the importance of *technology* as a separate category has been confirmed. Advancements in ICT are seen as necessary to improve the effectiveness of mobile and remote work, as they affect all key characteristics of work design. New technologies are needed to promote job autonomy, provide feedback, enable varied tasks, and improve work–life integration. In particular, the development of tools that improve virtual meetings is seen as relevant. Virtual spaces and the integration of artificial intelligence promise to simplify the provision of relevant data and improve social exchange (Kauffeld and Sauer 2021). Accordingly, organizations need to be aware that the improvement of virtual and hybrid meetings is a key to effective virtual collaboration.

In summary, the work scenarios can be used to identify future developments in work and reflect on them in terms of the associated opportunities and risks. Thus, this tool will help scholars find suitable solutions for organizations that incorporate employees’ needs and ponder the role and design of teamwork.

### Limitations

Some limitations of this study need to be addressed. First, the pre-determined number of Delphi rounds represents a methodological limitation. Even though many studies have employed a predefined number of Delphi rounds (Diamond et al. 2014), this procedure contradicts a basic principle of the method. As a rule, the assessment of the scenarios must reach a high degree of stability for Delphi studies to be completed (Rowe and Wright 2001). Several studies, however, have shown that repeated rounds of questioning might result in participants’ exhaustion, potentially leading to higher drop-out rates (Keeney et al. 2001; Rowe and Wright 2001). A limitation in terms of content can be seen in our focus on work in general. This study takes the first step toward evaluating the future of a post-COVID-19 world of work. Accordingly, we concentrated on the classification and structuring of scenarios. Future research should broaden the perspective by analyzing the effects of the pandemic on different branches of industry. Significant differences could be expected in office work, industrial production, and the service sector, among others. As our sample consisted of German-speaking participants only, cultural factors might have influenced our results in terms of the derived scenarios as well as the assessment of their desirability or probability. Thus, future research should also consider possible cross-cultural differences. Another limitation is found in the scope of our scenarios since we based the scenarios on recent developments in work. Along with the dramatic changes due to the pandemic, however, new trends have emerged, such as presentism (Kinman and Grant 2020). Future research should therefore monitor new trends in mobile work and their integration into the work design framework.

## Conclusion

Our study contributes to the research on the future of work, as it provides knowledge about desirable and probable work scenarios in a post-COVID-19 world. The digital transformation is continuing to advance, with mobile work and virtual collaboration growing steadily. This progress promotes to advantages on the organizational and individual levels, while disadvantages are mainly relating to teamwork. Specifically, positive developments are expected for technology (e.g., advancements in virtuality and artificial intelligence), leadership (e.g., increase in shared leadership and participation), and work–life integration (e.g., more flexibility and self-management). In contrast, the negative effects seen for teamwork include team cohesion and social exchange becoming more difficult to build and maintain. The identified opportunities and risks highlight the relevance of work design in the context of the ongoing digital transformation. Therefore, companies need to be aware of the impact of mobile and remote work on task, knowledge, social, and contextual characteristics to effectively shape the future of work.
